# Functional diversity of supragranular GABAergic neurons in the barrel cortex

**DOI:** 10.3389/fncir.2012.00052

**Published:** 2012-08-17

**Authors:** Luc J. Gentet

**Affiliations:** Cognitive and Systems Neuroscience Group, Swammerdam Institute for Life Sciences, Universiteit van AmsterdamAmsterdam, Netherlands

**Keywords:** inhibition, GABAergic, interneuron, somatosensory, cortex, circuit

## Abstract

Although the neocortex forms a distributed system comprised of several functional areas, its vertical columnar organization is largely conserved across areas and species, suggesting the existence of a canonical neocortical microcircuit. In order to elucidate the principles governing the organization of such a cortical diagram, a detailed understanding of the dynamics binding different types of cortical neurons into a coherent algorithm is essential. Within this complex circuitry, GABAergic interneurons, while forming approximately only 15–20% of all cortical neurons, appear critical in maintaining a dynamic balance between excitation and inhibition. Despite their importance, cortical GABAergic neurons have not been extensively studied *in vivo* and their precise role in shaping the local microcircuit sensory response still remains to be determined. Their paucity, combined with their molecular, anatomical, and physiological diversity, has made it difficult to even establish a consensual nomenclature. However, recent technological advances in microscopy and mouse genetics have fostered a renewed interest in neocortical interneurons by putting them within “visible” reach of experimenters. The anatomically well-defined whisker-to-barrel pathway of the rodent is particularly amenable to studies attempting to link cortical circuit dynamics to behavior. To each whisker corresponds a discrete cortical unit equivalent to a single column, specialized in the encoding and processing of the sensory information it receives. In this review, we will focus on the functional role that each subtype of supragranular GABAergic neuron embedded within such a single neocortical unit may play in shaping the dynamics of the local circuit during somatosensory integration.

## Introduction

When the whisker of a rodent contacts an object, the ensuing deflection is encoded into action potential output of sensory neurons located in the infraorbital branch of the trigeminal nerve. These neuronal signals eventually reach the primary somatosensory cortex, where the information they contain is thought to be integrated and processed [reviewed in Petersen (2007)]. While transfer of information within the brain is achieved through the activation of excitatory synapses, inhibition is required to maintain a crucial and dynamic balance within local circuits. This inhibition is mediated by the activation of local GABAergic interneurons releasing the neurotransmitter gamma aminobutyric acid (GABA) onto their postsynaptic targets. A basic view of GABAergic cell function is that inhibition curbs recurrent local excitation which would otherwise lead to runaway circuit activity (Moore et al., 2010). Indeed, in most cells, inhibition is temporally encoded to coincide with excitation (Okun and Lampl, 2008). In neuronal modeling, only one type of cell is generally sufficient to achieve such an effect. Why then does the neocortex contain such a diverse family of GABAergic cell subtypes?

One answer lies in the anatomical and computational complexity of principal excitatory cells themselves. While neocortical pyramidal neurons receive thousands of excitatory inputs, many are located at sites too far away from the axon initial segment (AIS) to have a significant impact on action potential generation. However, coincident and temporally precise sequences of dendritic inputs may generate local Ca^2+^ spikes that efficiently propagate to the soma and initiate action potential firing (Williams and Stuart, 2002; Larkum et al., 2007; Branco et al., 2010). Some GABAergic neurons have adapted to this architectural complexity by targeting their axon terminals to localized areas of the somatodendritic arbors of pyramidal cells (PC), hence the terms perisomatic inhibition, dendrite-targeting interneurons and axo-axonic cells. Further groups of GABAergic cells target mainly other interneurons, or display long-range projections throughout the neocortex (Staiger et al., 2004; Tomioka et al., 2005). The ability to dynamically and locally modulate the excitatory drive received by principal cells may impart the circuit with increased computational power, necessary for the efficient integration of sensory input.

The diversity of cortical GABAergic neurons extends beyond targeted domains of principal cells, and it is now widely accepted that molecular and physiological properties also contribute to this remarkable heterogeneity (Cauli et al., 1997; Markram et al., 2004; Yoshimura and Callaway, 2005; Lu et al., 2007; Ascoli et al., 2008; Burkhalter, 2008). Ideally, GABAergic subtypes have unique molecular, physiological, and functional properties that clearly distinguish themselves from each other. However, overlap in chemical marker composition and age-dependent variability in electrophysiological properties often lead to confusing results between studies. Interestingly, almost all GABAergic interneurons in the mouse somatosensory cortex can be accounted for on the basis of three non-overlapping chemical markers whose expression is developmentally-regulated: neurons from the medial ganglionic eminence (MGE) express either the calcium-binding protein parvalbumin (PV) or the neuropeptide somatostatin (Sst) (Rudy et al., 2011). Similarly, neurons emanating from the caudal ganglionic eminence (CGE) express the inonotropic serotonin receptor 5HT3a (5HT3aR) (Lee et al., 2010). Here, we rule out the possibility of a continuum of GABAergic cellular subtypes, but we also caution against strict separations without proof of functional relevance.

It is reasonable to assume that the diversity of cortical GABAergic neurons has functional implications beyond simply curbing local excitation. Recently, attempts to uncover the functional correlates of this diversity within the rodent somatosensory cortex *in vivo* have been successful in assigning precise roles to specific subtypes of cells in the regulation of the local circuit activity (Cardin et al., 2009; Gentet et al., 2012). These advances have been made possible by recent technological developments in microscopy, optogenetics, and mouse transgenesis. Today, researchers have all the necessary tools to uncover the precise functions of further supragranular GABAergic cell subtypes by combining the strengths of these available tools.

## Overview: available tools

Traditional *in vivo* methods of neuronal activity recordings such as microelectrodes or blind-patching seldom report the activity of GABAergic populations due to their paucity in the neocortex. Extracellular single-unit recordings from the neocortex usually differentiate only two types of neurons based on their waveform characteristics: regular-spiking excitatory principal cells and fast-spiking (FS) inhibitory neurons. Non-FS GABAergic neurons, which form the majority of cortical interneurons, may therefore often be mistaken for pyramidal neurons without proper identification. Analytical tools such as cross-correlation of spike dynamics provide increasingly better resolution, but errors in properly assigning single-unit recordings to a particular cellular type are still unavoidable (Barthó et al., 2004).

In order to unequivocally and efficiently record the neuronal activity of GABAergic neurons in the intact brain, we require modern optical and genetic tools that allow the *visualization* and therefore, the *targeting* of cellular subtypes of interest.

Fortunately, these are now becoming widely available, thanks to efforts by several researchers to generate genetically-engineered mouse strains targeting cortical GABAergic neurons. These transgenic mice models provide access to different subtypes of inhibitory neurons with ever-increasing specificity through the expression of fluorescent proteins, thus allowing the visualization of subclasses of interest. While 10 years ago, one had access to only a few mouse models, such as the GAD67-GFP mouse (Tamamaki et al., 2003) or the GIN mouse (Oliva et al., 2000), today dozens of mice lines are available for experimental purposes (López-Bendito et al., 2004; Taniguchi et al., 2011, see figures for a list of models available for each cortical GABAergic neuron subtype). As a cautionary note, it must be mentioned that the inherent genetic diversity of cortical GABAergic neurons implies that most mouse models should be used merely as a tool to narrow down one's search for a functionally relevant subtype. For example, while the VIPCre mouse model is best suited for identifying and/or manipulating bipolar cells (BPCs), a small subset of basket cells (BCs) also express vaso-intestinal polypeptides (Gupta et al., 2000), together with potentially a variety of other interneurons (Rudy et al., 2011).

The next requirement is to use optical tools that allow the visualization of the fluorescent cells. While epifluorescence or confocal microscopy might be sufficient for experimenters using slice preparation, more sophisticated equipment is needed to access cortical inhibitory neurons in the intact brain. Non-linear two photon-excited fluorescence microscopy (2P microscopy) allows cellular imaging several hundred microns deep in living animal tissue (Helmchen and Denk, 2005) and is ideally suited for the study of supragranular GABAergic neuron activity.

Finally, one must carefully choose a method of recording or manipulating the neuronal activity of these cells, depending on one's needs. Here, we will briefly describe some of the useful techniques that a researcher may decide upon.

## *In vitro* electrophysiology

While studying the behavior-mediated functional role of any cell type within a circuit requires a live animal, *in vitro* studies can shed valuable light on the wiring diagram and passive properties of different types of cortical GABAergic neurons. While more details of intra- and interlaminar inhibitory connectivity are beginning to emerge (Xu and Callaway, 2009; Fino and Yuste, 2011; Packer and Yuste, 2011; Avermann et al., 2012), a complete map of subtype-specific interneuronal connectivity within a cortical column is still lacking. To better understand the functional role played by cortical GABAergic neurons in somatosensory information processing, it is essential to know what their precise presynaptic and postsynaptic neuronal partners are within the local circuit. Through detailed anatomical and electrophysiological analysis of their connectivity and synaptic properties, it can be determined whether a particular type of interneuron may be involved in basic circuit algorithms such as feedforward, feedback, lateral inhibition or disinhibition (Gupta et al., 2000; Porter et al., 2001; Hull et al., 2009).

## *In vivo* calcium-imaging

This is a powerful technique for obtaining information pertaining to the suprathreshold activity of many neurons simultaneously [reviewed in Grienberger and Konnerth (2012)]. While certain injected calcium dyes will stain all neurons around the injection site, it is possible to isolate GABAergic populations of interest by carefully choosing appropriate mouse models [for example expressing tdTomato fluorescent proteins instead of GFP (green fluorescent protein) when using Oregon Green BAPTA-1 indicators, (Runyan et al., 2010)]. Genetically-engineered calcium indicators now enable long-lasting recordings of neuronal activity, allowing the incorporation of learning paradigms in experimental procedures (for example, see Huber et al., 2012). However, it is unclear how the overall higher firing rates of cortical GABAergic cells affect the quality and temporal precision of calcium signals.

## *In vivo* cell-attached electrophysiology

An easier alternative to whole-cell electrophysiology, this technique only requires the close proximity of the electrode tip to a cell. Fluorophore-coated glass pipettes may help in carefully positioning the tip in order to avoid mechanical disturbance of the soma (Ishikawa et al., 2010). This recording configuration only allows the observation of neuronal spikes but can lead to long-lasting valuable *in vivo* recordings of GABAergic cell suprathreshold activity with high temporal precision (for example, see Ma et al., 2010). Cell-attached or loose-patch recordings have also been used as control experiments aiming to directly related calcium signals with AP output or as a proof of principle of the efficacy of viral expression in optogenetics experiments (see below). Finally, the close proximity of the pipette tip with the neuronal membrane allows single-cell electroporation and the delivery of viral vectors for cell labeling or neuronal network tracing (Marshel et al., 2010). If experimental conditions are not sufficiently optimized, cell-attached recordings may however lead to erroneous firing rates being reported (Alcami et al., 2012).

## *In vivo* whole-cell electrophysiology

Targeted intracellular recordings allow the observation of single-cell membrane potential dynamics with submillisecond and submillivolt precision (Margrie et al., 2003). This technique can be applied to obtain information about the membrane potential dynamics of GABAergic neurons in head-fixed, yet awake, behaving animals (Gentet et al., 2010). *In vivo* whole-cell recordings appear particularly useful in the light of the sparse firing regime of the neocortex, which indicates that most of the electrical activity occurring in principal cells is in the range of potentials below the spiking threshold. This technique, when combined with a nearby local field potential or dual whole-cell recordings can shed valuable light on the involvement of GABAergic cells in cortical oscillations (Gentet et al., 2012). Furthermore, through the inclusion of biocytin within the pipette solution, full morphological reconstructions of the somatodendritic and axonal arborizations of recorded neurons can be achieved. However, whole-cell recordings lead to a washout of the intracellular milieu and often suffer from poor access resistance.

## Optogenetics

This powerful technique relies on the light-responsive properties of certain ion-permeable microbial opsins which can be expressed in mammalian neurons. Among these, channelrhodopsin-2 (ChR2) is a cation channel which, upon blue light stimulation, leads to rapid depolarization of the neuronal membrane potential and reliable action potential firing. On the other hand, halorhodospin (NpHR) is a chloride pump that is activated upon yellow light illumination, leading to rapid hyperpolarization of the neuronal membrane potential and suppression of action potential firing. Co-expressed with conditional AAV expression vectors carrying transgene cassettes that are activated only in the presence of Cre, these opsins can be targeted for expression in specific cell types using a variety of commercially-available Cre-driver mice lines (Taniguchi et al., 2011; Madisen et al., 2012). However, since there are no mouse lines specifically targeting supragranular GABAergic neurons, localized viral injections combined with *post-hoc* anatomical verification are required to spatially restrict opsin expression to those cells under investigation. For activation or inactivation of supragranular GABAergic neurons, LEDs can be an affordable light sources for reliable stimulation (Campagnola et al., 2008). Optogenetics is particular useful in obtaining causal links between the modulation of the activity of specific interneurons and its impact on network dynamics in principal cells, from which one can better infer their function (Royer et al., 2012).

## Subtypes of supragranular cortical neurons

While a precise categorization of cortical inhibitory neurons remains elusive, we have chosen to focus on eight different subtypes of cells, two of which are located in layer 1 (L1) and six of which in layer 2/3 (L2/3). Because of their intrinsic connectivity patterns, chemical marker content, anatomical features and passive electrophysiological properties, these eight different types of cells may form functionally-relevant clusters of inhibitory sources within local microcircuits. GABAergic cells of a particular subtype most often form electrical synapses or gap junctions between themselves, a property that may promote synchronization of action potential discharges [reviewed in Hestrin and Galarreta (2005)]. Cell-type specific gap junctions further enhance the segregation of cortical GABAergic neurons into distinct subtypes.

Some of the following neuronal classes have already been studied *in vivo*, while the behaviorally-dependent neuronal activity of others still remains to be determined. Here, we provide a framework for engaging the research community into probing still deeper into this fascinating family of cortical neurons.

## Layer 1

**Figure 1:**
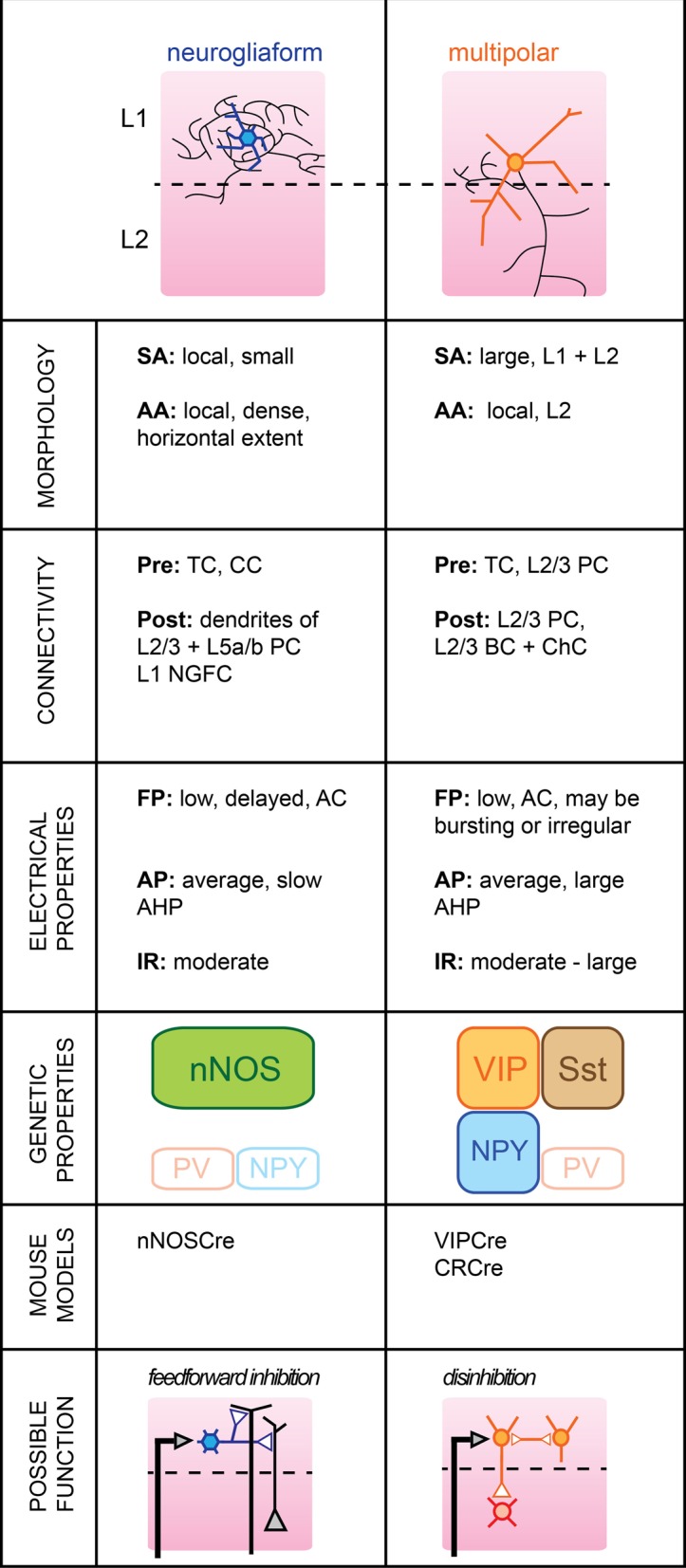
**Subtypes of cortical GABAergic neurons in layer 1. Top**: schematic representation of neuronal morphology. Thick colored lines: somatodendritic arborization; thin black lines: axonal arborization. Abbreviations per row. Morphology, **SA**, somato-dendritic arborization; **AA**, axonal arborization. Connectivity, **Pre**, presynaptic source; **Post**, postsynaptic target; **TC**, thalamocortical afferents; **CC**, corticocortical afferents; **PC**, pyramidal cell; **NGFC**, neurogliaform cell; **BC**, basket cell; **ChC**, chandelier cell. Electrical properties, **FP**, firing pattern; **low**, <100 Hz maximal firing rate; **moderate**, 100–150 Hz; **high**, >150 Hz; **AC**, accommodating; **AP**, action potential; **AHP**, after-hyperpolarization; **IR**, input resistance. Genetic properties (filled top boxes: expressed markers; bottom empty boxes: markers not expressed), **nNOS**, neuronal nitric oxide synthase; **NPY**, neuropeptide Y; **PV**, parvalbumin; **VIP**, vasoactive intestinal polypeptide; **Sst**, somatostatin. **Bottom**: schematic representation of possible functional role within the local circuit. Black arrows indicate excitatory inputs; reversed white triangles represent inhibitory inputs.

This outermost layer (“molecular layer”) of the cortex extends roughly 10% of the way down at the level of mouse somatosensory cortex. Almost all neurons located within its boundaries are believed to be GABAergic in the mature cortex (Hestrin and Armstrong, 1996). L1 also contains the apical dendrites of both L2/3 and L5 PC, which are thought to underlie cross-areal top-down interactions in cortical networks and it has been shown that activation of L1 neurons *in vivo* powerfully modulates sensory responses in L2/3 PC (Shlosberg et al., 2006). Despite being often overlooked, L1 clearly plays a significant role in information processing within a column of barrel cortex. Here, we will focus on the two major types of mature GABAergic neurons identified so far in L1 and indicate potentially interesting lines of research that one could pursue in order to investigate their respective functional role within the local cortical circuit (Figure 1).

## Neurogliaform cells

Neurogliaform cells (NGFCs) form one of two major subtypes of GABAergic neurons identified in the outermost layer of barrel cortex. Genetically, they are characterized by the expression of neuronal nitric oxide synthase (nNOS) (Kubota et al., 2011), while anatomically, they form dense local axonal arborizations that often extend horizontally (Hestrin and Armstrong, 1996; Chu et al., 2003).

In brain slices, L1 NGFCs were found to form gap junctions with each other in 80% of pairs tested and chemical synapses in one fourth of pairs tested (Chu et al., 2003). They may receive glutamatergic inputs from both thalamocortical or corticocortical afferents terminating in L1 (Cauller et al., 1998), but do not appear to be contacted by L2/3 PCs (Wozny and Williams, 2011, but see Chu et al., 2003). In turn, their dense local axonal arborizations suggest a large innervation of L2/3 and L5a/b PCs, possibly extending to nearby columns. Inhibitory postsynaptic potentials mediated by NGFC firing notably include a slow GABA_B_ component in addition to GABA_A_-mediated responses (Palmer et al., 2012).

Their membrane properties include a low to moderate input resistance level, a late-spiking regime of near-threshold discharge and a fast after-hyperpolarization (AHP). The level of spike frequency adapation during high current injections was found to be non-accommodating in the juvenile rat (Chu et al., 2003) but accommodating in the more mature neocortex (Wozny and Williams, 2011).

L1 NGFCs express nNOS but, unlike their L2/3 counterparts are not thought to express neuropeptide Y (NPY) (Karagiannis et al., 2009). Thus, they form a neuronal population distinct from L2/3 NGFC.

L1 NGFCs have a large receptive field, responding with short latency to a small deflection of the principal whisker (Zhu and Zhu, 2004). Because NGFC cells activate slow metabotropic GABA_B_ receptors, possibly by volume transmission (Oláh et al., 2009), they could provide long-lasting inhibition at the level of both local L2/3 and L5a/b PC apical dendrites (Figure 1). We speculate that activation of L1 NGFCs may ensure homeostatic maintenance of dendritic inhibition when the animal engages in active somatosensory behaviors by replacing the tonic inhibition provided by L2/3 Martinotti cells (MCs) during quiet wakefulness (Gentet et al., 2012). In order to address this issue, it will be useful to record the activity of L1 NGFCs in awake animals where active whisking behavior may be observed.

The horizontal extent of their axons also suggests a possible function in time-dependent lateral inhibition of surrounding columns. Corticocortical afferents terminating in S1 may be non-specific, and the lateral inhibition provided by NGFCs could allow the gating of top-down inputs in a column-specific manner.

## Multipolar cells

The second major GABAergic cell subtype present in L1 has a large multipolar dendritic tree and a descending axon which can reach as far as layer V (Chu et al., 2003).

While one study found that Multipolar cells (MpCs) can respond to principal whisker deflection with short latencies, indicating that they may receive direct thalamocortical afferents (Zhu and Zhu, 2004), further confirmation is required as MpCs may be mistaken for atypical layer 2 PCs (Van Brederode et al., 2000). MpCs may receive glutamatergic projections from higher cortical areas (Cauller et al., 1998), as well as cholinergic inputs from the basal forebrain (Mechawar et al., 2000; Arroyo et al., 2012). Some MpCs receive excitatory inputs from local L2/3 PCs (Wozny and Williams, 2011). Their postsynaptic targets include L2/3 PCs and, more interestingly, L2/3 FS cells, possibly both BCs and Chandelier cells (ChCs) (Christophe et al., 2002; Letzkus et al., 2011).

Various firing patterns of MpCs have been reported previously; MpCs with deep-layer projecting axons can respond in a burst-like mode of AP discharge (Wozny and Williams, 2011) with little accommodation, while others report an irregular but adapting spiking pattern upon current pulse injection (Chu et al., 2003; Arroyo et al., 2012). Input resistance was generally higher in MpCs than in L1 NGFC neurons.

A subset of MpC neurons express calretinin (CR) in addition to alpha-actinin-2 (Aac) which is also present in L1 NGFCs (Lee et al., 2010; Kubota et al., 2011). They may also express vasoactive intestinal polypeptide (VIP) and Sst, although this remains to be confirmed (Xu et al., 2010; Arroyo et al., 2012). The lack of specific molecular markers for MpCs suggests that they may represent a more diverse family of interneuronal subtypes that will require further subclassification in future studies.

MpCs may possess a large receptive field but postsynaptic responses from passive deflections of surrounding whiskers were found to be dramatically reduced compared to responses from principal whisker deflections (Zhu and Zhu, 2004). Recently, it was shown that L1 interneurons in auditory cortex could be activated by aversive stimuli, in turn leading to inhibition of L2/3 PV-positive cells (Letzkus et al., 2011). Whether a similar disinhibitory mechanism takes place in rodent somatosensory cortex remains to be determined. Nevertheless, it appears that MpCs may be involved in rapid inhibition of BCs and/or Chandelier neurons in supragranular layers. Most FS cells display reduced firing rate during active whisking behavior, but their membrane potentials remained unaffected (Gentet et al., 2010). Shunting inhibition emanating from L1 MpCs could account for this reduction in neuronal excitability.

## Layer 2/3

**Figure 2:**
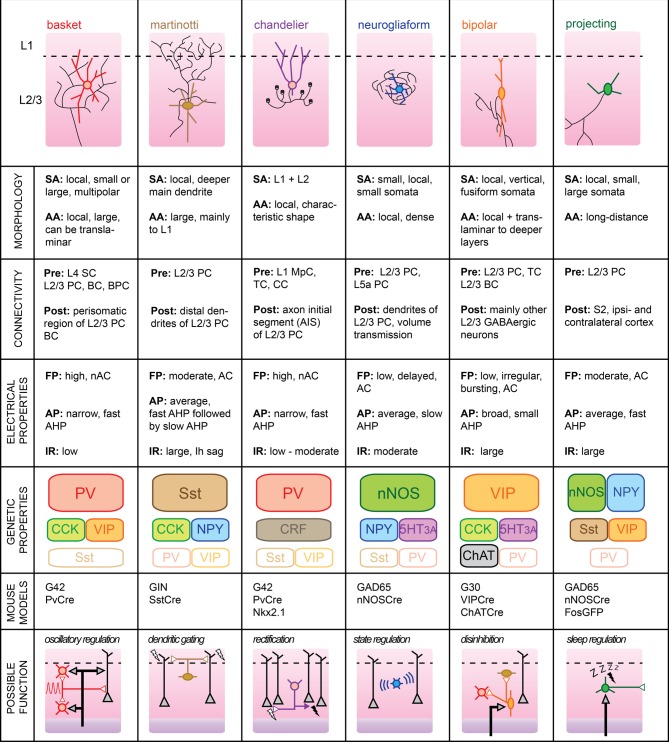
**Subtypes of cortical GABAergic neurons in layer 2/3. Top**: schematic representation of neuronal morphology. Thick colored lines: somatodendritic arborization; thin black lines: axonal arborization. Abbreviations per row. Morphology, **SA**, somato-dendritic arborization; **AA**, axonal arborization. Connectivity, **Pre**, presynaptic source; **Post**, postsynaptic target; **SC**, Spiny stellate cell; **PC**, pyramidal cell; **BC**, basket cell; **BPC**, bipolar cell; **TC**, thalamocortical afferents; **CC**, corticocortical afferents; **MpC**, multipolar cell. Electrical properties, **FP**, firing pattern; **low**, <100 Hz maximal firing rate; **moderate**, 100–150 Hz; **high**, >150 Hz; **AC**, accommodating; **nAC**, non-accommodating; **AP**, action potential; **AHP**, after-hyperpolarization; **IR**, input resistance. Genetic properties (filled top boxes: expressed markers; middle boxes: markers expressed in subsets; bottom empty boxes: markers not expressed), **PV**, parvalbumin; **Sst**, somatostatin; **NPY**, neuropeptide Y; **VIP**, vasoactive intestinal polypeptide; **CCK**, cholecystokinin; **CRF**, corticotropin-releasing factor; **nNOS**, neuronal nitric oxide synthase; **5HT3A**, serotonin receptor 3A; **ChAT**, choline acetyltransferase. **Bottom**: schematic representation of possible functional role within the local circuit. Black arrows indicate excitatory inputs; reversed white triangles represent inhibitory inputs.

Supragranular L2/3 of barrel cortex are the main recipients of excitatory inputs from the input layer 4 of a functional cortical column (Lübke et al., 2000). While there are no cytoarchitectural differences between the two layers (hence, their combined name), some studies indicate that local PC have different inputs/output functions dependent upon their cortical depth (Shepherd and Svoboda, 2005; Lefort et al., 2009). However, it is still unclear whether L2 and L3 differ functionally. Most L2/3 pyramidal neurons fire sparsely during somatosensory-guided behaviors, indicating that inhibition is likely to play a major role in shaping their activity (Brecht, 2007). In the mouse somatosensory cortex, cortical GABAergic cells form approximately 15% of the total neurons located in L2/3 (Lefort et al., 2009). A more specific “hot zone” with higher GABAergic cell density was recently found in layer 2 of rat somatosensory cortex (Meyer et al., 2011). The large variety of GABAergic neuron subtypes embedded within the local microcircuit is likely to dynamically control the integration of somatosensory signals taking place at the level of supragranular layers. Here, we will review the properties of six identified subgroups of GABAergic L2/3 cells and attempt to formulate functional roles which, in most cases, still remain to be validated (Figure 2).

## Basket cells

BCs form the largest group of L2/3 cortical GABAergic neurons. As such, BC interneurons are thought to provide the main source of inhibition within the local circuit. Their name stems from the basket-like appearance of the innervation they provide around the somata of L2/3 PC. In studies using extracellular recording techniques, BCs are often simply referred to as “FS” cells, based on their fast AP waveform. Subdivisions of this large family of cortical GABAergic interneurons have also been proposed, based on the variable sizes of their somatodendritic arborizations and extents of their axonal plexus (Wang et al., 2002). For example, so-called “large BCs” could provide lateral inhibition within the somatosensory cortex by extending their axons into neighboring columns (Helmstaedter et al., 2009).

BCs mainly target the perisomatic area of local pyramidal neurons with the formation of several boutons from distinct axonal branches per postsynaptic cell (Chattopadhyaya et al., 2004). Recently, it was shown that a single L2/3 BC can innervate most of its neighboring PCs within a 100 μm radius in mouse somatosensory cortex (Packer and Yuste, 2011). In turn, BCs receive excitatory inputs from both L2/3 PCs and L4 spiny stellate cells (SCs) (Helmstaedter et al., 2008; Matéo et al., 2011). While BCs might preferentially target local PC, they also efficiently innervate other L2/3 BCs and, to a lesser extent, other local interneurons (Tamás et al., 1998; Gibson et al., 1999; Avermann et al., 2012). Finally, BCs are believed to form networks of electrically connected cells through cell-type specific Cx36-containing gap junctions that allow synchronization of their activity (Galarreta and Hestrin, 1999; Deans et al., 2001).

BCs typically express PV and calbindin (CB), but can also co-express a variety of other neuropeptides including NPY, VIP, and cholecystokinin (CCK) (Druga, 2009; Karagiannis et al., 2009).

Upon current pulse injection, BCs can display the highest firing rates of all cell types, with no accommodation of spike frequency. APs elicited in these cells are of brief durations and are followed by large but rapid AHP potentials. *In vivo*, the spontaneous firing rate of BC neurons was found to be the highest amongst all L2/3 cell types despite their low input resistance and high rheobase (Gentet et al., 2010). We speculate that their low intrinsic excitability is counterbalanced by their extensive connectivity with local excitatory neurons. Indeed, BCs exhibit suprathreshold responses with high reliability upon passive whisker deflection, presumably because they are well innervated by L4 SCs (Gentet et al., 2012).

Because of their rapid kinetics and high levels of intra- and interconnectivity with L2/3 PC neurons, BCs are ideally suited for the *regulation of oscillations* within the local circuit (Holmgren et al., 2003). In particular, they are believed to be involved in gamma (30–80 Hz) oscillations, which are thought to enable fast processing and binding of distributed responses (Fries et al., 2007). Indeed, stimulation of PV-immunoreactive interneurons (which also includes ChCs, a caveat not to be overlooked) using optogenetics is capable of inducing gamma rhythms in mouse somatosensory cortex (Cardin et al., 2009). In a further study in mouse prefrontal cortex, it was shown that abolishment of their activity suppresses spontaneous gamma oscillations (Sohal et al., 2009). While short bouts of gamma oscillations can be recorded in local field potentials from granular and infragranular layers of barrel cortex during sensory stimulation and anticipatory activities in unrestrained rats (Jones and Barth, 1997; Hamada et al., 1999), supragranular layers typically exhibit reduced gamma activity but rather display slower rhythms, both during quiet wakefulness and during active whisking (Crochet and Petersen, 2006). In the future, it will therefore be essential to determine whether L2/3 BCs also participate in the regulation of these oscillations.

Since L2/3 BCs are likely to be the most prevalent source of GABAergic inhibition impinging on L2/3 PCs, their synchronized activation is likely to play a major role in shaping sensory responses in a cortical column of rodent somatosensory cortex (Cardin et al., 2009). In the visual cortex, L2/3 BCs were found to modulate cortical gain by dramatically altering the spiking responses of local PC to visual stimuli while leaving their basic tuning properties unchanged (Atallah et al., 2012). Whether a similar functional role can be assigned to BCs of the barrel cortex during active whisking remains to be determined.

Recently, it was shown that horizontally-projecting L2/3 PCs can impose layer-specific lateral inhibition through the recruitment of inhibitory circuits in neighboring columns (Adesnik and Scanziani, 2010). Whether a morphologically distinct subgroup of the BC family mediates this circuit mechanism remains to be determined. Recordings of such neurons in adjacent columns of a sensory-driven principal whisker column may help uncover this specific functional role.

## Martinotti cells

L2/3 MCs distinguish themselves by an extensive axonal arborization in L1 and a promiscuous innervation of L2/3 pyramidal neurons (Wang et al., 2004; Fino and Yuste, 2011). Despite their low numbers in a typical somatosensory column, this dense and unspecific connectivity places them in an ideal position to control the general dendritic excitability of local excitatory cells.

L2/3 MCs receive facilitatory synaptic inputs from local pyramidal neurons. Two PC neurons and one intermediate MC can thus constitute the basic elements of a frequency-dependent disynaptic inhibitory subcircuit (Silberberg and Markram, 2007). MCs rarely form chemical synapses with each other and may therefore rather receive inhibitory inputs from other GABAergic subtypes, such as BPCs (Staiger et al., 2004) or deeper inhibitory neurons from the granular layer 4 (Xu and Callaway, 2009).

The passive membrane properties of MCs include a large input resistance and a low rheobase which, combined with a more depolarized resting membrane potential than other L2/3 cortical neurons, renders them particularly excitable (Fanselow et al., 2008). Their relatively narrow APs are followed by two AHP potentials with different kinetics leading to a complex triphasic waveform (Ma et al., 2006). Finally, injection of hyperpolarizing current reveals the presence of I_h_ channels in the shape of a distinctive sag in the membrane potential.

The main molecular blueprint of MCs is the presence of Sst. Subsets of Sst cells in the somatosensory cortex may express the calcium-binding proteins CB or CR, indicating that several classes of Sst-containing interneurons may co-exist (Halabisky et al., 2006). It is unclear whether this molecular diversity has any functional correlate in the more mature brain.

Recently, it was shown that *in vivo* activity of MCs is characterized by a high rate of discharge during quiet wakefulness which is dramatically reduced during active whisker sensorimotor processing (Gentet et al., 2012). Optogenetic inhibition of their activity led to an increase in burst firing in neighboring pyramidal neurons, mirroring a marked increase in L1 dendritic calcium activity during whisking. Thus, it appears that MCs act as an “ON-switch” in mouse somatosensory cortex, effectively shunting the distal dendrites of the local excitatory L2/3 pyramidal neurons when the mouse is not engaged in somatosensory processing, but rapidly releasing their tonic inhibition when the animal engages in exploratory behavior.

In the future, it will be interesting to uncover the synaptic source of likely GABAergic inhibition that leads to the membrane hyperpolarization of MCs during active whisking and active touch (Gentet et al., 2012, see “BPCs” section).

## Chandelier cells

Also known as “axo-axonic cells,” ChCs represent a rare subtype of PV-containing GABAergic interneurons that uniquely target the AIS of local pyramidal excitatory cells (Inda et al., 2009). They derive their name from their distinctive axonal arborizations terminated by short vertical bundles climbing upwards along the AIS of their postsynaptic targets and which resemble the candlesticks of a chandelier (Somogyi et al., 1982; Figure 2).

L2/3 ChCs often display fusiform somata with dendrites extending upwards parallel to the apical dendrites of local pyramidal neurons. While the cellular source of their presynaptic inputs remains to be determined, the primary extent of their dendrites into L1 (especially for ChC neurons located in layer 2, see Woodruff et al., 2011) suggests that they may receive direct thalamocortical and corticocortical afferents. Since passive whisker stimulation evokes an early IPSP in L2/3 ChCs in the anesthetized rat (Zhu et al., 2004), we speculate that ChCs may receive inhibitory inputs originating from L1 MpCs known to target L2/3 FS neurons (Christophe et al., 2002). The axons of ChCs neurons mainly extend downwards before ramifying sideways, and then protruding back upwards where they form up to 12 boutons on potentially 250 L2/3 PC targets (Somogyi et al., 1982). Thus, despite their low numbers, L2/3 ChCs cells are ideally positioned to directly control the output of a vast number of local excitatory neurons within their respective functional cortical column.

*In vitro*, passive membrane properties of ChCs are often indistinguishable from those of BCs in terms of resting membrane potential, input resistance, AP waveform, or membrane spike threshold. However, careful analysis of the subthreshold current-voltage relationship of ChCs neurons compared to BCs has shown that the former possess a linear I/V plot while the latter invariably display non-linearities (Woodruff et al., 2009). Nevertheless, neuronal anatomical reconstruction through dye-filling will still remain the most convincing proof that one has indeed recorded from a ChC.

Recently, the inhibitory nature of these cortical GABAergic neurons has been questioned. Under recording conditions designed not to disrupt the intracellular milieu of patched neurons, it was shown that AP initiation in a presynaptic ChC could lead to a depolarizing potential in a postsynaptic PC (Szabadics et al., 2006; Woodruff et al., 2011). This effect was attributed to the the differential distribution of KCCN transporter subtypes along the axo-somato-dendritic axis of PC neurons leading to a more depolarized GABA_A_ reversal potential in the AIS (Khirug et al., 2008). Estimates for this axonal E_GABA_ place it between the average membrane potentials corresponding to UP- and DOWN- states of the local network. The intriguing possibility that ChCs may have an excitatory impact remains controversial and was recently challenged by a study showing that all interneurons, including axo-axonic cells, hyperpolarized hippocampal pyramidal neurons (Glickfeld et al., 2009). However, if E_GABA_ is indeed more depolarized in cortical L2/3 PCs, the impact of ChC activation on sensory integration in L2/3 PCs would be dependent upon the overall membrane potential level of the locally-synchronized excitatory neurons (Petersen et al., 2003). Upon passive whisker deflection, ChC neurons would depolarize their postsynaptic targets during periods of DOWN states, while inhibiting them during UP-states, thus acting as “rectifiers” of the local circuit activity. On the other hand, during active whisking, ChC activity should in fact be shunting, as the Vm level of L2/3 PCs approaches that of axonal E_GABA_ (Crochet and Petersen, 2006).

Since perforated-patch clamp recordings *in vivo* seem difficult to envisage, a combination of whole-cell recordings of ChC neurons together with calcium-imaging of neighboring L2/3 PCs appear to be the most promising avenue for establishing the true functional impact of this cortical GABAergic subtype in shaping the activity of the local circuit during somatosensory integration.

## Neurogliaform cells

L2/3 NGFCs are morphologically characterized by small somata and short but numerous dendrites extending radially. Similarly, their axonal plexus is spatially restricted but densely branched, leading to the formation of numerous presynaptic boutons in close proximity of each other (Karube et al., 2004). Thus, NGFCs form an “axonal cloud” within which they may exert a powerful inhibitory control. Recently, it was shown that this anatomical configuration confers NGFCs with the ability to impact any neuronal compartment located within their spheres of influence through a process called “volume transmission.” This form of non-synaptic communication relies on high levels of neurotransmitter release, such that sufficient GABA reaches extrasynaptic GABA_A_ receptors located on neuronal membranes (Oláh et al., 2009). Finally, NGFC neurons can elicit slow inhibitory responses in L2/3 PCs through activation of metabotropic GABA_B_ receptors (Tamás et al., 2003).

The connectivity pattern of NGFCs is not well characterized. While they receive excitatory inputs from local L2/3 PCs and possibly L5a PCs (Xu and Callaway, 2009), it is unclear whether they can also receive direct thalamocortical, corticocortical, or L4 excitatory inputs. NGFC neurons have been shown to elicit slow IPSPs in a variety of interneurons on top of L2/3 PCs (Oláh et al., 2007). They are also the only known type of cortical GABAergic cells that form electrical synapses with other interneuronal subtypes (Simon et al., 2005). Therefore, NGFCs seem ideally suited to monitor the sub and suprathreshold activity of the local network.

Cortical NGFCs express nNOS and may be involved in neurovascular coupling through release of NO which acts as a vasodilator (Cauli and Hamel, 2010). Contrary to their L1 counterparts, L2/3 NGFCs may express NPY (Karagiannis et al., 2009) and ionotropic serotonin receptors (5HT3A) (Vucurovic et al., 2010). Therefore, NGFC neurons may be activated by ascending neuromodulatory inputs.

NGFCs respond to depolarizing current pulses with late spiking APs with moderate half-widths followed by a slow AHP. Current injections of increasing amplitudes reveal an accommodating pattern of AP discharge (Oláh et al., 2007). Input resistances of NGFCs as measured *in vitro* fall within the average range of values for GABAergic neurons.

The functional role of NGFCs in the local circuit of the rodent barrel cortex remains to be determined. The ability of NGFCs to monitor the activity of many neurons within its axonal cloud through electrical synapses and inhibit them through volume transmission indicates a potentially powerful influence of this GABAergic subtype during physiologically relevant sensory processes. Are NGFCs responsible for the slow phase transitions between depolarized UP- and resting DOWN-states observed *in vivo*? Blocking GABA_B_ receptors in a model of Up-Down states elicited in rat slices increases the duration of depolarized network activity (Mann et al., 2009). Does *in vivo* local pharmacological blockade of these receptors lead to similar disruption of network activity? Alternatively, an investigation of the *in vivo* membrane potential dynamics of NGFC neurons might reveal a preferential discharge during phase transitions to DOWN-states of the slow synchronous fluctuations. Finally, the ability of NGFCs to tonically activate extrasynaptic δ-subunit-containing GABA_A_ receptors suggests their possible involvement in stress-related modulation of sensory integration (Mody and Maguire, 2011).

## Bipolar cells

L2/3 BPCs mostly have a fusiform somatodendritic arborization, with two main opposing dendrites arranged in a vertically-orienting fashion (Bayraktar et al., 2000). Typically, the horizontal spread of the somatodendritc arborization is limited, while the vertical spread may encompass both L1 and L5b.

The firing pattern of BPC interneurons upon current pulse depolarization can be very diverse, ranging from regular-spiking, almost pyramidal cell-like, to irregular, with an initial burst of APs followed by accommodating spikes (Cauli et al., 2000; Lee et al., 2010). As measured *in vitro*, their input resistance appears to be relatively large compared to other cortical GABAergic neurons, indicating that they may be highly excitable (Karagiannis et al., 2009; Vucurovic et al., 2010).

BPCs receive excitatory inputs from local pyramidal neurons (Porter et al., 1998; Caputi et al., 2009) and may be targets of direct thalamocortical inputs (Staiger et al., 1996; Lee et al., 2010). PV-immunoreactive boutons were found to innervate BPCs, indicating that they may receive functionally-relevant inhibitory inputs from local BCs (Staiger et al., 1997). BPCs appear to specifically target other types of local GABAergic cells (Dávid et al., 2007; Vucurovic et al., 2010).

BPC neurons express VIP as well as CR but usually are not immunoreactive for PV and rarely express CB. They may also express CCK, choline acetyltransferase (ChAT), serotonin 3a receptors (5HT3AR), making them the most genetically diverse subtype of supragranular cortical GABAergic neuronal classes (Ferezou et al., 2002; Lee et al., 2010). Their fast excitation by serotonergic fibers also raises the possibility of their involvement in mood-related circuit modulation (Ferezou et al., 2002).

A potential function for BPCs within the local circuit is to regulate the activity of other types of cortical GABAergic cells through *disinhibition*, judging from previous indirect evidence in the hippocampus and rat barrel cortex (Staiger et al., 2004; Klausberger and Somogyi, 2008). Specifically, are BPCs the source of GABAergic inputs hyperpolarizing MCs during whisking (Gentet et al., 2012)? To uncover this role, it will be necessary to record their activity during active whisking with high temporal precision, in order to establish whether they are indeed capable of providing a reliable source of hyperpolarizing potentials to MCs. A first clue that this might be the case comes from the observation that non-FS GABAergic cells, to which BPCs belong, can dramatically increase their firing rates during whisking (Gentet et al., 2010). Optogenetic silencing of BPC neurons during whisking (which may be attained using the VIPCre mouse model) should then allow MCs to discharge persistently, thus precluding encoding of sensory information arriving at distal dendrites of L2/3 PCs. Finally, if BPC neurons reliably increase their firing coincidentally with the initiation of whisker motion, it would be interesting to find out whether they receive direct excitatory inputs emanating from the primary motor cortex (Petreanu et al., 2009).

## Projecting cells

Type I nNOS-containing neurons account for only 0.5–2% of all cortical GABAergic cells, making them the rarest clearly separable subgroup, but also the least well characterized. They distinguish themselves from other cortical GABAergic neurons by their long-range axonal projections, which may extend as far as contralaterally (Tomioka et al., 2005). Thus, they are not strictly speaking interneurons, but rather GABAergic long-range projecting neurons (LPNs).

LPNs heavily label for nNOS, and most cells also express NPY and Sst. It is assumed that any cell expressing all of these three markers will fall within this GABAergic subtype (Kilduff et al., 2011). Interestingly, CB was found to be present in LPNs in the rat somatosensory cortex, but absent in the same region of mouse (Gerashchenko et al., 2008). While they are present in most layers, their density increases with cortical depth (Perrenoud et al., 2012).

The electrophysiological properties of LPNs are not well characterized due to their paucity. One study combining *post-hoc* RT-PCR of whole-cell recorded neurons points toward a large input resistance and a fast AHP following moderately-broad, accommodating, APs (Karagiannis et al., 2009).

Interestingly, a population of nNOS-positive cortical cells with similar genetic characteristics as LPNs was identified as being sleep-active using Fos immunohistochemistry (Gerashchenko et al., 2008). Due to their ability to project to many distant targets within the neocortex, it has been suggested that these specialized GABAergic neurons may provide a basis for homeostatic sleep regulation (Kilduff et al., 2011). It remains to be determined through careful electrophysiological recordings whether LPNs truly are specifically active during sleep and what the impact of this activity implies for neocortical network regulation.

## Other GABAergic cell types

While “double-bouquet” cells have a distinctive axonal morphology in the shape of a descending horsetail, indicating that they could therefore form a functional GABAergic subtype of their own, their presence in rodent somatosensory cortex has been challenged (Yañez et al., 2005, but see Zhu et al., 2004). Furthermore, their genetic characteristics are not well understood and thus, no mouse model can be proposed to specifically target this class of cells.

A third subtype of PV-containing interneuron termed “multipolar bursting cells (MBCs)” has been identified in juvenile mouse neocortex *in vitro* (Blatow et al., 2003). There were invariably located in L2 close to the laminar border with L1 and displayed a bursting pattern of activity upon current pulse injection distinct from BCs or ChCs. It is unclear whether these differences are a result of developmental maturation of the circuit and the presence of MBCs in the adult rodent brain still remains to be confirmed.

## Conclusion and outlook

In this review, we have focused on the potential functional role played eight of the numerous cellular subtypes encountered within the family of cortical GABAergic supragranular neurons. While we assigned one possible circuit role to each of these, it is probable that any particular GABAergic subtype we described conveys several key functional properties to the cortical network in a state-dependent manner. In order to better understand how each subtype is integrated into the local circuit dynamics, it will be essential to monitor their activity during different behaviors. The whisker of a rodent may be passively deflected, or a rodent may engage in active whisking behavior leading to large and rapid cycles of whisker protractions and retractions interrupted when a nearby obstacle is encountered. Preliminary results suggest that GABAergic neurons are differentially activated during such behavioral events (Gentet et al., 2010; Crochet et al., 2011), leading to the possibility that specific inhibitory microcircuits may be responsible for the processing of somatosensory information during different behaviors of the animals (Yoshimura and Callaway, 2005).

While most cortical GABAergic cells are interneurons with restricted local axonal arborizations, their recruitment may involve presynaptic neurons from many parts of the brain. Ascending pathways from the brainstem are particularly prone to such cell-type specific neuromodulation. Behaviorally-relevant fast synaptic excitation of specific cortical GABAergic subtypes through the activation of inotropic 5HT3AR receptors may be controlled by cortically-projecting serotonergic neurons in the dorsal raphe (Bang et al., 2012). Direct GABAergic and cholinergic projections emanating from the basal forebrain are capable of modulating the activity of different groups of cortical GABAergic cells (Freund and Meskenaite, 1992; Disney and Aoki, 2008). Finally, histaminergic pathways involved in the regulation of the sleep-wake cycle form axonal terminals in primary somatosensory cortex, although their GABAergic neuronal targets still remain to be determined (Bayer et al., 2004). Future research into the impact of neuromodulation on sensory processing will need to take into account the likely involvement of specific local GABAergic neurons.

Recently, the recruitment of cortical GABAergic neurons has been established in the cortico-cortical crossmodal synaptic inhibition of L2/3 PC in mouse primary sensory cortices (Iurilli et al., 2012). While a translaminar inhibitory circuit has been hypothesized to be responsible for this effect, the exact nature of the cortical GABAergic neurons was not further investigated.

We live an exciting time in the field of cortical GABAergic neuron research. More powerful and specific transgenic mice models are bound to emerge in the next few years, offering researchers opportunities to probe with ever greater details the array of complex functions subserved by this essential neuronal family. In the future, we should endeavor to perform more systematic studies of the response properties of identified GABAergic neurons to help us uncover the temporal sequence of synaptic events leading to the efficient sensory integration of somatosensory information within a single barrel column. This basic framework for sensory processing may subsequently serve in the formulation of rules governing a putative canonical neocortical circuit [reviewed in Douglas and Martin (2004)] that may be applied to other sensory modalities, and to humans.

### Conflict of interest statement

The author declares that the research was conducted in the absence of any commercial or financial relationships that could be construed as a potential conflict of interest.
